# The co-inhibitory molecule PD-L1 contributes to regulatory T cell-mediated protection in murine crescentic glomerulonephritis

**DOI:** 10.1038/s41598-018-38432-3

**Published:** 2019-02-14

**Authors:** Katrin Neumann, Annett Ostmann, Philippe Christophe Breda, Aaron Ochel, Frank Tacke, Hans-Joachim Paust, Ulf Panzer, Gisa Tiegs

**Affiliations:** 1 Institute of Experimental Immunology and Hepatology, University Medical Center Hamburg-Eppendorf, Hamburg, Germany; 2 Department of Medicine III, RWTH-University Hospital Aachen, Aachen, Germany; 3 III. Medical Clinic, University Medical Center Hamburg-Eppendorf, Hamburg, Germany

## Abstract

Immune-mediated glomerular diseases like crescentic glomerulonephritis (cGN) are driven by inappropriately regulated cellular and humoral immune responses subsequently leading to renal tissue injury. Recent studies demonstrated the crucial role for regulatory T cells (Tregs) in suppressing pathogenic T-cell responses during nephrotoxic nephritis (NTN), a murine model of cGN. However, mechanisms of immune regulation in cGN are less clear. Here, we aim at investigating the role of the co-inhibitory PD-1/PD-L1 pathway in Treg-mediated suppression of renal inflammation. We demonstrated that Foxp3^+^ Tregs expressing PD-L1 infiltrate the kidney during NTN. Inhibition of PD-L1 signalling by using *PD-L1*^−*/*−^ mice or by blockage of PD-L1 in wildtype mice resulted in an increased Treg frequency in the inflamed kidney. However, mice lacking PD-L1 developed more severe NTN associated with an elevated pathogenic renal Th1 immune response, which was reversed by blockage of IFNγ in these mice. Interestingly, lack of PD-L1 altered the gene expression profile of Tregs in homeostasis and kidney inflammation. Functionally, Tregs from nephritic *PD-L1*^−*/*−^ mice had impaired suppressive capacity *in vitro* and failed to protect from NTN *in vivo*. Thus, PD-L1 displays a protective role in NTN, which is related to Treg-mediated suppression of the Th1 immune response.

## Introduction

Crescentic glomerulonephritis (cGN) is a severe glomerular disease characterized by formation of glomerular crescents in Bowman’s space and a rapid loss of renal function. Inappropriately regulated cellular and humoral immune responses, which may result from defects in central and peripheral tolerance, drive cGN. Negative co-stimulatory pathways are crucial for the maintenance of peripheral tolerance by inducing inhibitory signals in lymphocytes. One negative co-stimulator receptor expressed on activated T cells and B cells is programed cell death-1 (PD-1) that is bound by programed cell death ligand-1 (PD-L1) and PD-L2. PD-L1 is expressed by hematopoietic and non-hematopoietic cells and can be further induced during inflammation. In contrast, PD-L2 expression is mostly restricted to activated dendritic cells (DCs) and macrophages^1,2^. The PD-l/PD-L1 pathway exerts important functions in immune regulation and promotes development and function of regulatory T cells (Tregs) by induction and maintenance of the Treg-specific transcription factor forkhead box protein P3 (Foxp3)^3^. Binding of PD-L1 to PD-1 during primary T-cell activation induces blockage of T-cell proliferation and cytokine production and inhibits cytotoxic activity and cell survival^4,5^. Moreover, effector T-cell reactivation and function is also negatively regulated by the PD-1/PD-L1 interaction^6,7^.

The PD-1/PD-L1 pathway has been implicated in immune regulation of kidney diseases. A single nucleotide polymorphisms in the PD-1 gene was associated with increased susceptibility of patients to systemic lupus erythematosus^8^. Moreover, aged *PD-1*^−*/*−^ mice were shown to develop lupus-like glomerulonephritis^9^. Renal expression of PD-L1 was demonstrated in patients with lupus nephritis, tubulointerstitial nephritis or renal cell carcinoma^10^. Furthermore, several studies revealed that blockage of PD-1/PD-L1 interaction aggravated murine accelerated nephrotoxic serum nephritis^11^, ischemia reperfusion-induced kidney injury^12^, adriamycin nephropathy^13^ or lupus-like nephritis^14^. However, mechanisms by which the PD-1/PD-L1 pathway mediates immunosuppression during kidney disease are less clear.

Kidney-infiltrating Th1 and Th17 cells were found to drive renal inflammation in murine models of cGN by production of the pro-inflammatory cytokines interferon-γ (IFNγ) and IL-17, respectively^15–19^. CD4^+^ Foxp3^+^ Tregs are crucial for the control of such pro-inflammatory immune responses to prevent excessive tissue damage and autoimmunity. We have shown recently that Tregs contribute to immune regulation in nephrotoxic nephritis (NTN), the murine model of cGN, by inhibiting the pro-inflammatory Th1 immune response thereby ameliorating disease pathogenesis^20^. The suppressive effect of Tregs during NTN was partially attributed to expression of the anti-inflammatory cytokine IL-10^21^. In the present study, we investigated the immunoregulatory role of the co-inhibitory PD-1/PD-L1 pathway in Treg-mediated protection from renal injury.

## Results

### Lack of PD-L1 resulted in an enhanced recruitment of Tregs into the inflamed kidney

**Figure 1:**
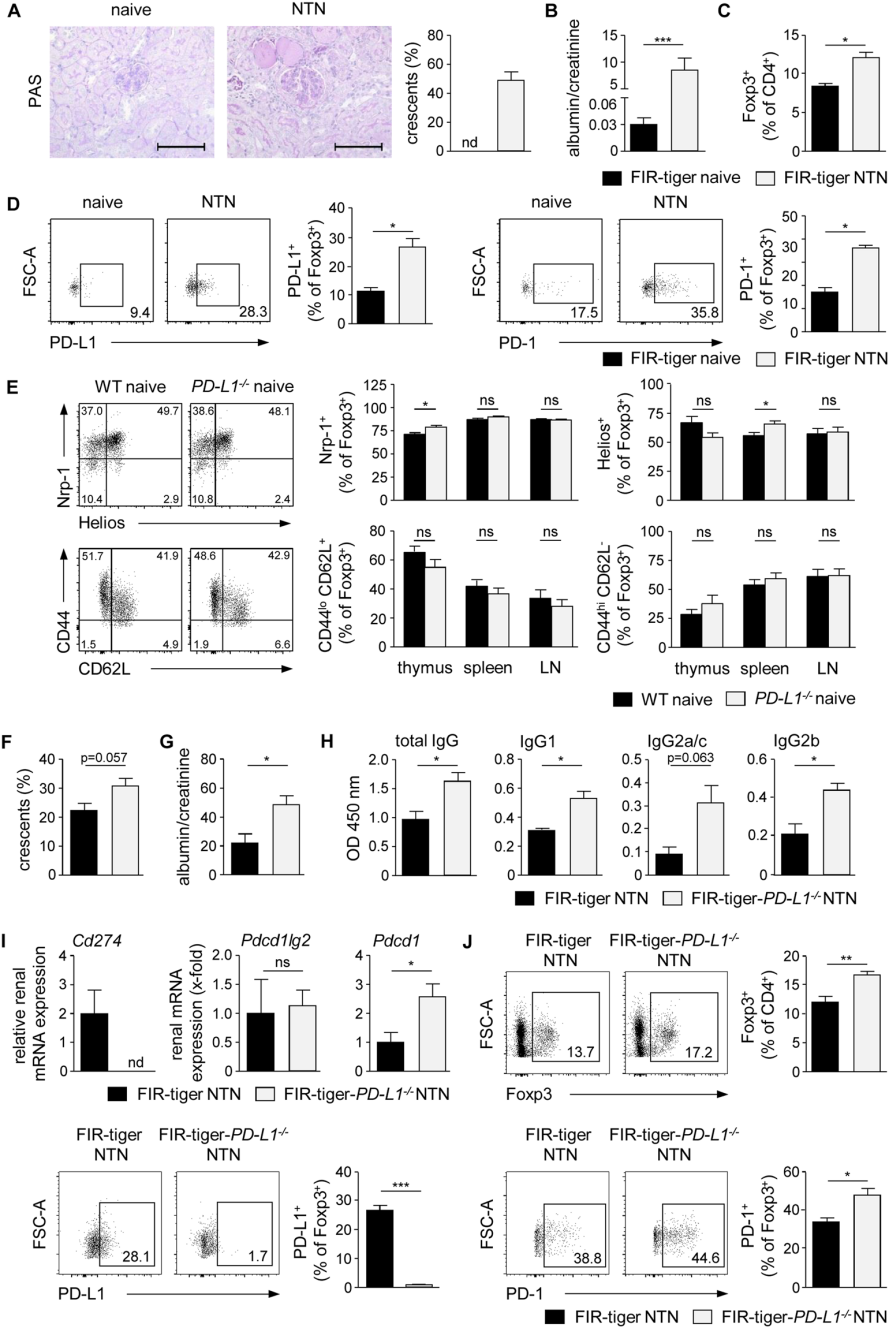
Increased renal recruitment of Tregs in the absence of PD-L1. (**A**) NTN was induced in FIR-tiger mice by i.p. injection of the nephritogenic NTN serum and mice were analyzed 8 days later. Glomerular crescent formation was quantified in PAS-stained kidney sections of naive and nephritic FIR-tiger mice. (**B**) Renal dysfunction was assessed by determination of the albumin-creatinine-ratio in urine by ELISA. (**C**) Frequencies of renal Foxp3^+^ Tregs and (**D**) PD-L1- or PD-1-expressing Foxp3^+^ Tregs were analyzed by flow cytometry in naive and nephritic FIR-tiger mice. (**E**) Expression of neuropilin-1 (Nrp-1), Helios, CD62L and CD44 was analyzed in Foxp3^+^ Tregs from thymus, spleen and lymph nodes (LN) of naive *PD-L1*^−*/*−^ and WT mice. (**F**) NTN was induced in FIR-tiger-*PD-L1*^−*/*−^ and FIR-tiger mice that were analyzed 8 days later. Crescent formation was quantified in PAS-stained kidney sections of nephritic mice. (**G**) Albumin-creatinine-ratio was determined in urine by ELISA. (**H**) Levels of mouse anti-sheep total IgG and isotypes of IgG1, IgG2a/c, and IgG2b were determined in serum of nephritic FIR-tiger-*PD-L1*^−*/*−^ and FIR-tiger mice by ELISA. (**I**) Renal mRNA expression of nephritic FIR-tiger-*PD-L1*^−*/*−^ mice was analyzed by quantitative real-time RT-PCR and normalized to mRNA expression of nephritic FIR-tiger mice. (**J**) Frequencies of Foxp3^+^ Tregs and PD-L1- or PD-1-expressing Foxp3^+^ Tregs were analyzed in nephritic FIR-tiger-*PD-L1*^−*/*−^ and FIR-tiger mice by flow cytometry. Representative photomicrographs and dot were shown. Scale bars represent 100 µm. Mean ± SEM of one experiment out of two experiments with 4 mice per group are shown. *p < 0.05; **p < 0.01; ***p < 0.001; ns, not significant; nd, not detectable; *Cd274*: PD-L1; *Pdcd1lg2*: PD-L2; *Pdcd1*: PD-1; *Il12b*.

The coinhibitory PD-1/PD-L1 pathway was found to contribute to Treg-mediated control of inflammatory immune responses. In this context, it was shown that *PD-L1*^−*/*−^ mice develop aggravated NTN and that lack of PD-L1 expression by cells of hematopoietic origin worsened disease pathogenesis^11^. Based on these finding, we asked whether Tregs might be responsible for PD-L1-mediated protection in NTN. Therefore, we induced NTN by injection of the nephritogenic NTN serum in FIR-tiger mice, which allow distinct detection of the Treg-specific transcription factor Foxp3 via flow cytometry^22^, and did Treg analysis in the T cell-mediated autologous phase 8 days after NTN induction. We analyzed glomerular damage by quantification of crescent formation in periodic acid-Schiff (PAS)-stained kidney sections^20^ and determination of proteinuria in urine by measurement of the albumin-creatinine-ratio. NTN serum-treated FIR-tiger mice developed severe NTN characterized by a high percentage of crescent formation and proteinuria whereas in naive FIR-tiger mice neither crescents nor proteinuria were detectable (Figs 1A,B). We showed an increased frequency of Foxp3^+^ Tregs in the inflamed kidneys of nephritic FIR-tiger mice compared to naive FIR-tiger mice (Fig. 1C, Supplemental Fig. 1A). Interestingly, the frequencies of renal Tregs expressing PD-L1 or its receptor PD-1 were strongly elevated in nephritic FIR-tiger mice (Fig. 1D) suggesting a function for the PD-1/PD-L1 pathway in Treg-mediated immune regulation in NTN.

To investigate the Treg response in absence of PD-1/PD-L1 signalling, we used *PD-L1*^−*/*−^ mice. As PD-L1 is involved in T-cell development and Treg induction^3,23^, we analyzed the Treg compartment in primary and secondary lymphoid organs of naive *PD-L1*^−*/*−^ mice. We excluded impaired generation of thymus-derived Foxp3^+^ Tregs since the frequencies of Helios^+^ and neuropilin-1^+^ Foxp3^+^ Tregs were comparable in *PD-L1*^−*/*−^ and C57BL/6 wildtype (WT) mice. Moreover, we found no substantial difference in the frequencies of CD44^lo^ CD62L^+^ central Tregs or CD44^hi^ CD62L^−^ effector Tregs (Fig. 1E, Supplemental Fig. 1B) indicating that lack of PD-1/PD-L1 signalling did not compromise the Treg compartment in naive *PD-L1*^−*/*−^ mice.

FIR-tiger mice were crossed with *PD-L1*^−*/*−^ mice to determine the renal Treg response during NTN in the absence of PD-L1 (Supplemental Figs 2, 3). We compared disease pathogenesis of NTN in FIR-tiger-*PD-L1*^−*/*−^ mice with the already known disease course in nephritic *PD-L1*^−*/*−^. In line with previous findings^11^ and our own data concerning *PD-L1*^−*/*−^ mice (Supplemental Fig. 4A,B), we demonstrated increased crescent formation and proteinuria in nephritic FIR-tiger-*PD-L1*^−*/*−^ mice compared to nephritic FIR-tiger mice (Fig. 1F,G). Further, the humoral immune response was analyzed by determination of the IgG antibody response against the nephritogenic antigen. In nephritic FIR-tiger-*PD-L1*^−*/*−^ mice, serum levels of mouse anti-sheep total IgG and of the isotypes IgG1, IgG2a/c, and IgG2b were enhanced compared to nephritic FIR-tiger mice (Fig. 1H). An elevated IgG antibody response was also determined in nephritic *PD-L1*^−*/*−^ mice (Supplemental Fig. 4C). Thus, lack of PD-L1 aggravated disease pathogenesis of NTN in both FIR-tiger-*PD-L1*^−*/*−^ mice and *PD-L1*^−*/*−^ mice.

We analyzed renal mRNA expression of PD-1 and its two ligands PD-L1 and PD-L2 during NTN. Nephritic FIR-tiger mice expressed PD-L1 (*Cd274*) mRNA, which was absent in FIR-tiger-*PD-L1*^−*/*−^ mice. We showed mRNA expression of PD-L2 (*Pdcd1lg2*) during NTN that was not altered in mice lacking PD-L1. In contrast, renal PD-1 (*Pdcd1*) mRNA expression was up-regulated in nephritic FIR-tiger-*PD-L1*^−*/*−^ mice compared to nephritic FIR-tiger mice (Fig. 1I). The same findings were shown in nephritic *PD-L1*^−*/*−^ mice. Moreover, PD-1 mRNA expression was even elevated in naive *PD-L1*^−*/*−^ mice compared to naive WT mice and was further enhanced during NTN (Supplemental Fig. 4D). Interestingly, we detected an increased frequency of Foxp3^+^ Tregs in the inflamed kidneys of FIR-tiger-*PD-L1*^−*/*−^ mice compared to nephritic FIR-tiger mice. The renal Treg population in nephritic FIR-tiger-*PD-L1*^−*/*−^ mice did not express PD-L1 and showed increased expression of PD-1 (Fig. 1J). Thus, recruitment of Foxp3^+^ Tregs into the inflamed kidney was increased in the absence of PD-L1.

### Up-regulated renal Th1 immune response in the absence of PD-L1

**Figure 2:**
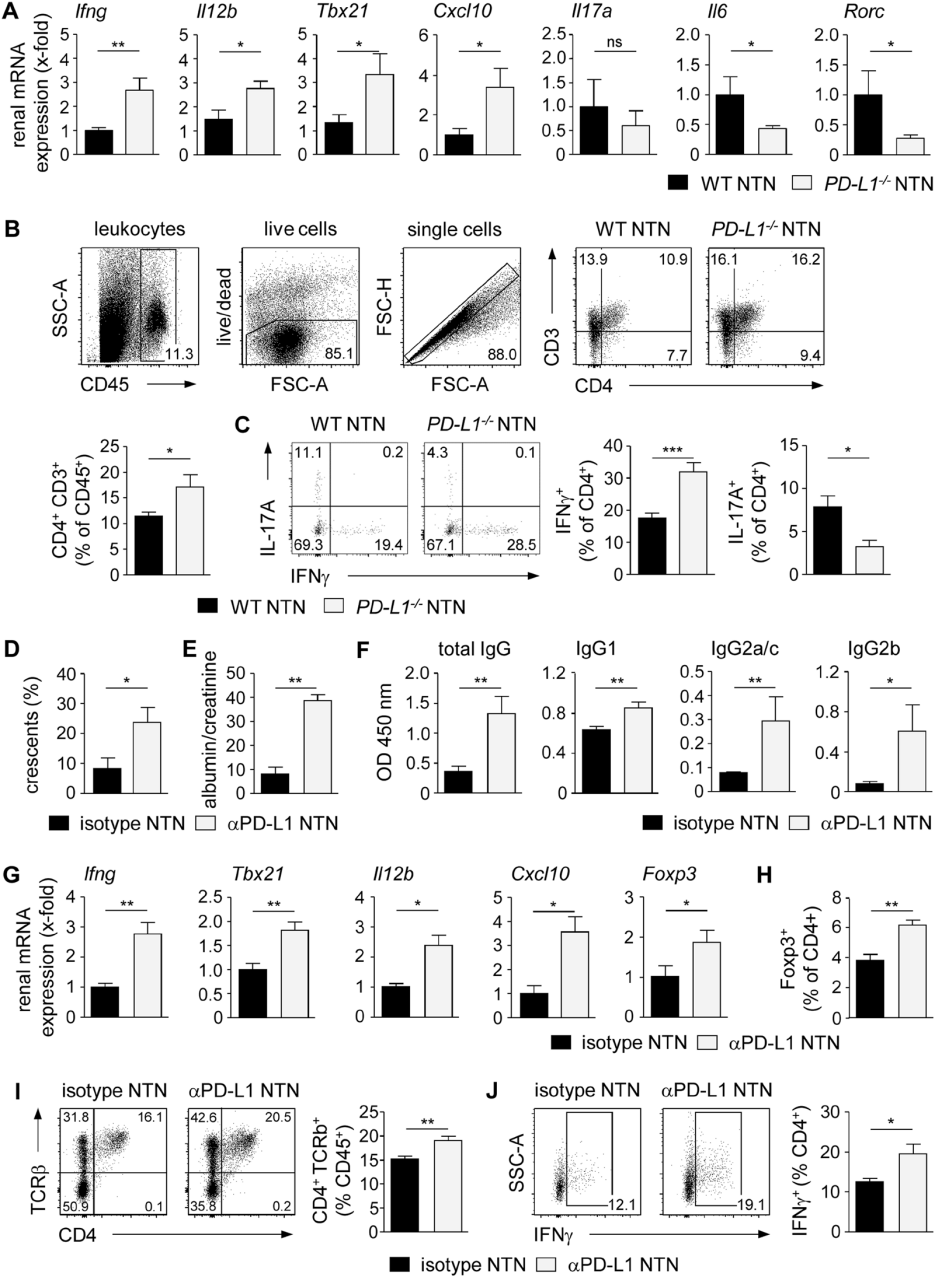
Increased frequency of renal IFNγ^+^ Th1 cells in nephritic *PD-L1*^−*/*−^ mice. (**A**) *PD-L1*^−*/*−^ and WT mice received i.p. NTN serum and were analyzed 8 days later. Renal mRNA expression of nephritic *PD-L1*^−*/*−^ mice was analyzed by quantitative real-time RT-PCR and normalized to mRNA expression of nephritic WT mice. (**B**) Frequency of renal CD4^+^ T cells was determined by flow cytometry. (**C**) Expression of IFNγ and IL-17A in renal CD4^+^ T cells of nephritic *PD-L1*^−*/*−^ and WT mice was analyzed by flow cytometry. Gating strategy and representative dot plots are shown. (**D**) WT mice received i.p. an anti-PD-L1 antibody or the respective isotype control and were analyzed 8 days after NTN induction. Crescent formation was quantified in PAS-stained kidney sections of anti-PD-L1 antibody-treated and isotype-treated nephritic WT mice. (**E**) Albumin-creatinine-ratio was determined in urine by ELISA. (**F**) Mouse anti-sheep IgG antibody levels were determined in serum by ELISA. (**G**) Renal mRNA expression of anti-PD-L1 antibody-treated nephritic WT mice was analyzed by quantitative real-time RT-PCR and normalized to mRNA expression of isotype-treated nephritic WT mice. (**H**) Frequencies of renal Foxp3^+^ Tregs, (**I**) CD4^+^ T cells, and (**J**) IFNγ^+^ CD4^+^ T cells were determined in anti-PD-L1 antibody-treated and isotype-treated nephritic WT mice by flow cytometry. Mean ± SEM of one experiment out of 2–3 experiments with 5–6 mice per group are shown. *p < 0.05; **p < 0.01; ***p < 0.001; ns, not significant; *Il12b*: IL-12 subunit p40; *Tbx21*: T-bet; *Rorc*: RORγt.

It has been shown that kidney-infiltrating Th1 cells and Th17 cells drive disease pathogenesis of NTN in WT mice^15–19^. Since Tregs were found to suppress Th1 cells during NTN^20^, we investigated the CD4^+^ T cell-mediated immune response in the absence of PD-1/PD-L1 signalling by using nephritic *PD-L1*^−*/*−^ mice. We analyzed renal mRNA expression and demonstrated elevated gene expression levels of the Th1-associated cytokines IFNγ (*Ifng*) and IL-12p40 (*Il12b*), and of the transcription factor T-bet (*Tbx21*) compared to nephritic WT mice. This correlated with enhanced expression of the IFNγ-inducible, Th1 cell-recruiting chemokine CXCL10 (*Cxcl10*) in nephritic *PD-L1*^−*/*−^ mice. In contrast, mRNA expression of genes associated with a Th17 cell-mediated immune response such as IL-6 (*Il6*) and RORγt (*Rorc*) was down-regulated (Fig. 2A). In homeostasis, renal gene expression of IFNγ, IL-12p40, T-bet, CXCL10, and IL-6 was not altered in naive *PD-L1*^−*/*−^ mice compared to WT mice whereas IL-17A and RORγt expression was not detectable (Supplemental Fig. 5A). We further investigated the local pro-inflammatory immune response by intracellular cytokine staining in renal T-cell populations. The frequency of CD4^+^ T cells was increased in the inflamed kidneys of nephritic *PD-L1*^−*/*−^ mice (Fig. 2B). In line with the gene expression data, we detected a significantly higher frequency of renal IFNγ-expressing Th1 cells in nephritic *PD-L1*^−*/*−^ mice than nephritic WT mice whereas the frequency of IL-17A-expressing Th17 cells was reduced. (Fig. 2C). We further showed that the frequency of Th1 cells did not differ in naive *PD-L1*^−*/*−^ mice and WT mice whereas Th17 cells were not detectable (Supplemental Fig. 5B).

We reinforced the findings in *PD-L1*^−*/*−^ mice by blockage of PD-L1 in WT mice. Therefore, WT mice were treated with an anti-PD-L1 antibody daily starting one day after induction of NTN. We showed that crescent formation, proteinuria, and humoral immune response were increased in anti-PD-L1 antibody-treated nephritic WT mice compared to isotype-treated nephritic WT mice (Fig. 2D–F). Renal mRNA expression of the Th1-associated molecules IFNγ, T-bet, IL-12p40, and CXCL10 was elevated after blockage of PD-L1 in WT mice. Interestingly, mRNA expression of Foxp3 was also increased (Fig. 2G) and this correlated with an enhanced frequency of renal Foxp3^+^ Tregs in anti-PD-L1 antibody treated nephritic WT mice compared to isotype-treated nephritic WT mice (Fig. 2H). We further detected an elevated frequency of CD4^+^ T cells and IFNγ^+^ Th1 cells in the kidneys of antibody-treated nephritic WT mice (Fig. 2I,J). Taken together, lack of PD-L1 resulted in an elevated renal Th1 immune response and aggravated NTN despite an increased frequency of Foxp3^+^ Tregs in the inflamed kidney.

### Augmented systemic Th1 immune response in the absence of PD-L1

**Figure 3:**
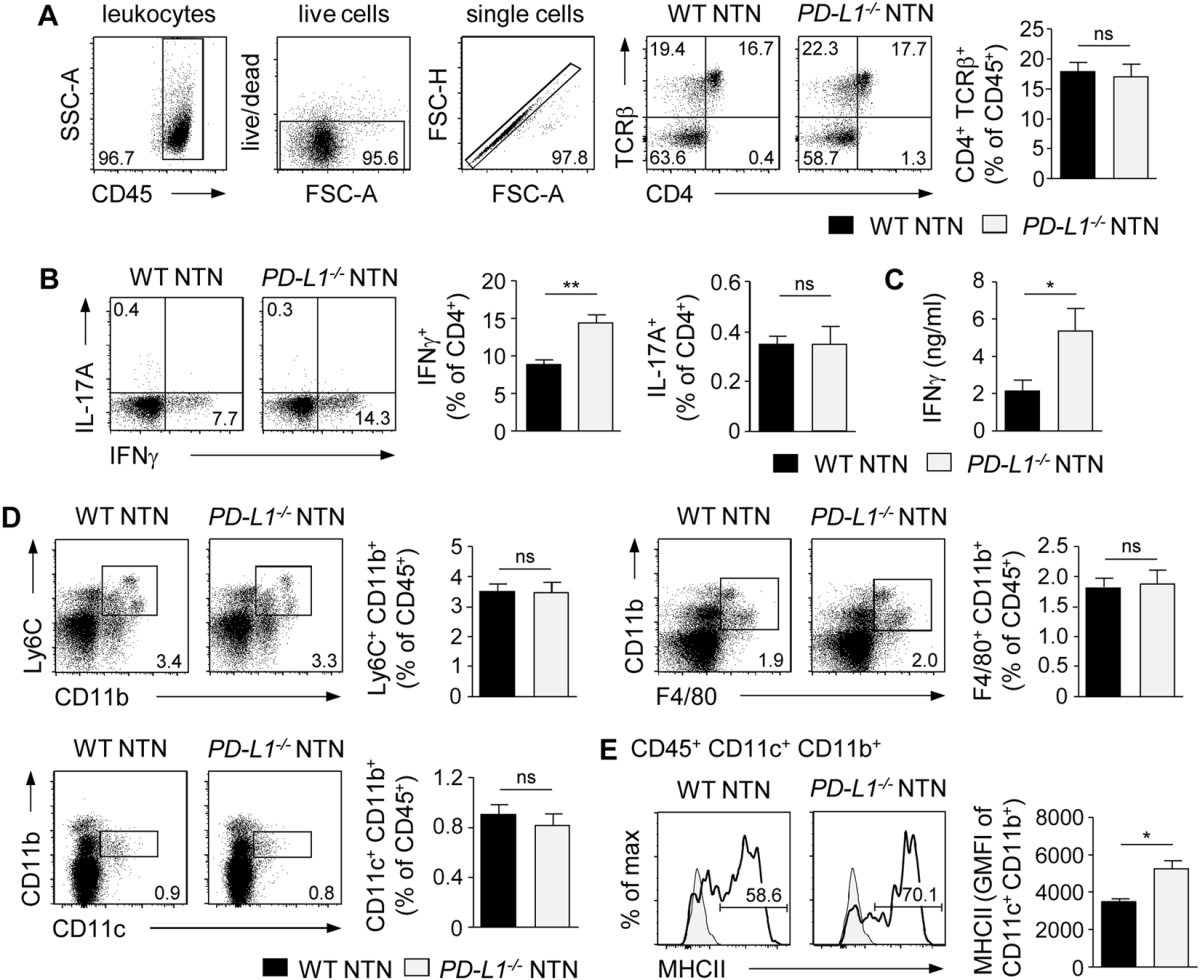
Enhanced systemic Th1 immune response in nephritic *PD-L1*^−*/*−^ mice. NTN was induced in *PD-L1*^−*/*−^ and WT mice that were analyzed 8 days later. (**A**) Frequency of splenic CD4^+^ T cells from nephritic *PD-L1*^−*/*−^ and WT mice was determined by flow cytometry. Gating strategy and representative dot plots are shown. (**B**) IFNγ and IL-17A expression was analyzed in splenic CD4^+^ T cells. (**C**) IFNγ levels in culture supernatants of sheep IgG-stimulated splenocytes from nephritic *PD-L1*^−*/*−^ and WT mice were measured by ELISA. (**D**) Frequencies of splenic Ly6C^+^ CD11b^+^ monocytes/macrophages, F4/80^+^ CD11b^+^ macrophages, and CD11c^+^ CD11b^+^ DCs from nephritic *PD-L1*^−*/*−^ and WT mice were determined. (**E**) Frequency and geometric mean fluorescent intensity (GMFI) of MHCII expressed by splenic DCs from nephritic *PD-L1*^−*/*−^ and WT mice was analyzed by flow cytometry. Representative dot plots and histograms are shown. Mean ± SEM of one experiment out of 2–3 experiments with 4–5 mice per group are shown. *p < 0.05; **p < 0.01; ns, not significant.

To investigate the role of PD-L1 regarding the systemic immune response during NTN, the phenotype of splenic CD4^+^ T cells was analyzed in nephritic *PD-L1*^−*/*−^ and WT mice. We found no alterations in the frequency of CD4^+^ T cells in nephritic *PD-L1*^−*/*−^ mice (Fig. 3A). However, we demonstrated an enhanced frequency of splenic IFNγ^+^ Th1 cells in the absence of PD-L1. In contrast, IL-17A was not substantially expressed by splenic CD4^+^ T cells during NTN (Fig. 3B). We further detected elevated IFNγ levels in culture supernatants of sheep IgG-stimulated splenocytes from nephritic *PD-L1*^−*/*−^ mice (Fig. 3C). We also analyzed splenic antigen-presenting cell populations that drive T-cell activation. No differences were detected in the frequencies of CD11b^+^ Ly6C^+^ monocytes/macrophages, F4/80^+^ CD11b^+^ macrophages or CD11c^+^ CD11b^+^ DCs in nephritic *PD-L1*^−*/*−^ and WT mice (Fig. 3D). Interestingly, the frequency of DCs expressing MHCII and their overall MHCII expression was increased in nephritic *PD-L1*^−*/*−^ mice (Fig. 3E).

### Ameliorated NTN after blockage of IFNγ in *PD-L1*^−*/*−^ mice

**Figure 4:**
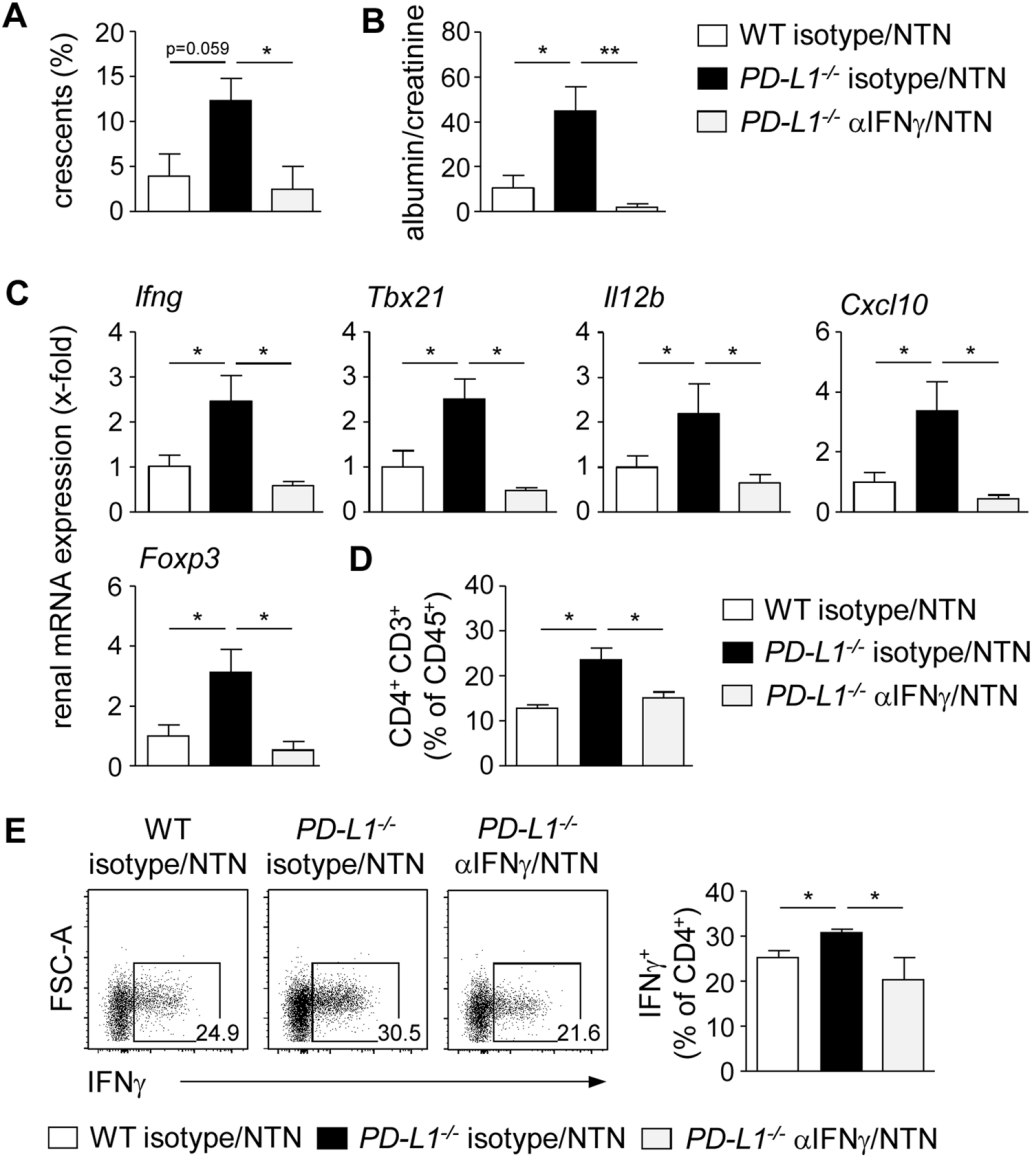
Blockage of IFNγ ameliorated NTN in *PD-L1*^−*/*−^ mice. *PD-L1*^−*/*−^ mice received i.p. an anti-IFNγ antibody or the respective isotype control and were analyzed 8 days after induction of NTN. (**A**) Crescent formation was quantified in PAS-stained kidney sections of anti-IFNγ antibody-treated nephritic *PD-L1*^−*/*−^ mice and isotype-treated nephritic *PD-L1*^−*/*−^ and WT mice. (**B**) Albumin-creatinine-ratio was determined in urine by ELISA. (**C**) Renal mRNA expression of anti-IFNγ antibody- and isotype-treated nephritic *PD-L1*^−*/*−^ mice was analyzed by quantitative real-time RT-PCR and normalized to mRNA expression of isotype-treated nephritic WT mice. (**D**) Frequencies of renal CD4^+^ T cells and IFNγ-expressing CD4^+^ T cells were analyzed by flow cytometry. Representative dot plots are depicted. Mean ± SEM of one experiment out of two experiments with 4 mice per group are shown. *p < 0.05; **p < 0.01; ns, not significant; *Il12b*: IL-12 subunit p40; *Tbx21*: T-bet.

Having shown that PD-L1 deficiency resulted in an increased IFNγ response and more severe kidney damage during NTN, we asked whether elevated IFNγ levels drive disease pathogenesis of nephritic *PD-L1*^−*/*−^ mice. Therefore, we blocked IFNγ by injection of a neutralizing anti-IFNγ antibody one day before and four days after induction of NTN in *PD-L1*^−*/*−^ mice and analyzed disease pathology. We demonstrated reduced crescent formation and proteinuria in anti-IFNγ antibody-treated nephritic *PD-L1*^−*/*−^ mice compared to isotype-treated nephritic *PD-L1*^−*/*−^ mice. Interestingly, disease severity of anti-IFNγ antibody-treated nephritic *PD-L1*^−*/*−^ mice was diminished to levels of isotype-treated nephritic WT mice (Fig. 4A,B). Renal mRNA expression analysis revealed strongly decreased expression of genes associated with a Th1 immune response such as IFNγ, T-bet, IL-12p40, and CXCL10 after neutralization of IFNγ in nephritic *PD-L1*^−*/*−^ mice. Moreover, Foxp3 mRNA expression was also decreased (Fig. 4C). In addition, the frequencies of renal CD4^+^ T cells and IFNγ^+^ Th1 cells were reduced in anti-IFNγ antibody-treated nephritic *PD-L1*^−*/*−^ mice (Fig. 4D,E). Taken together, blockage of IFNγ led to reduced renal infiltration of Th1 cells and ameliorated NTN in *PD-L1*^−*/*−^ mice.

### Altered gene expression profile of Tregs in the absence of PD-L1

**Figure 5:**
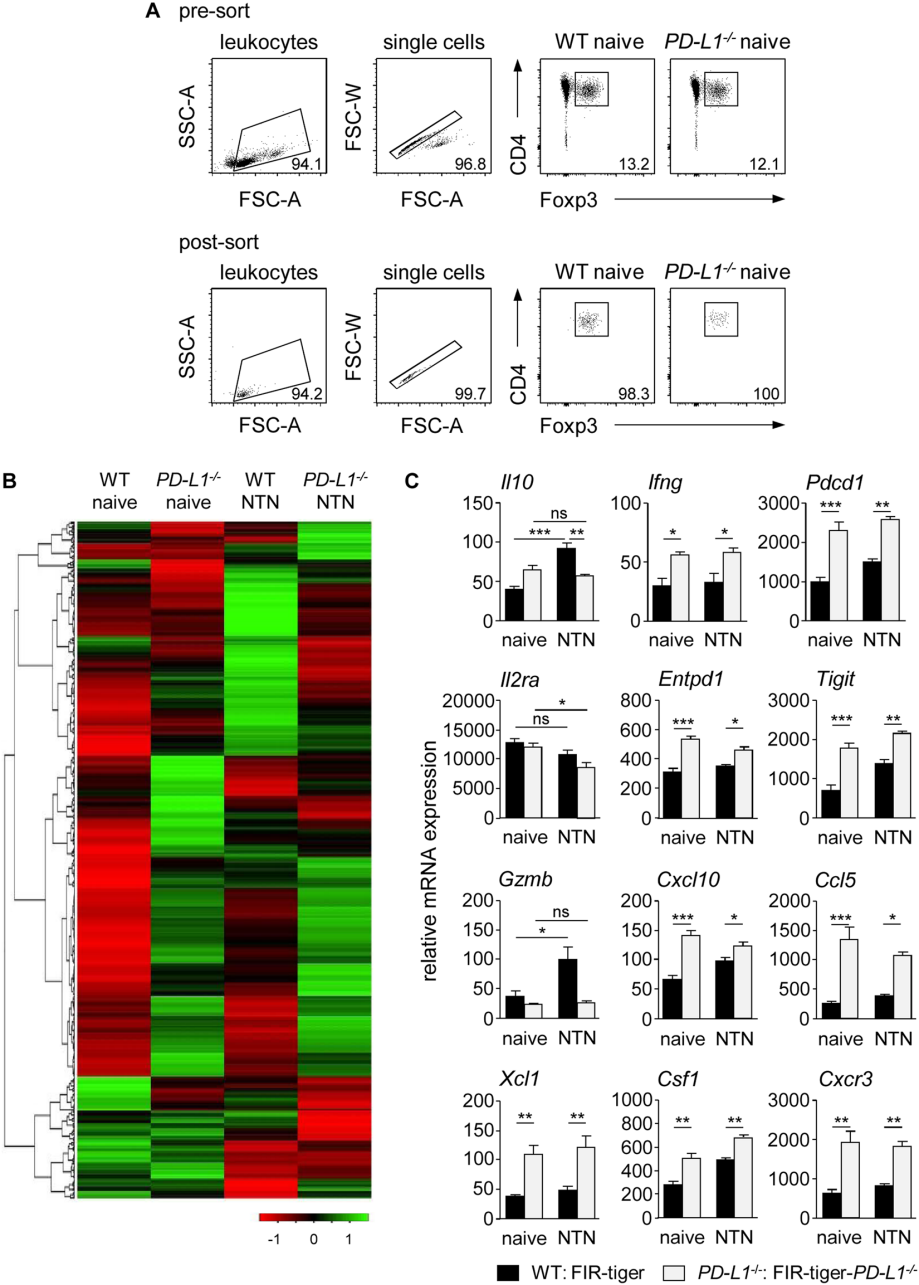
Lack of PD-L1 altered gene expression profile of Tregs. (**A**) Foxp3^+^ Tregs were isolated by FACS from naive and nephritic FIR-tiger-*PD-L1*^−*/*−^ and FIR-tiger mice. Representative dot plots show sorting strategy and purity of isolated Tregs. (**B**) NanoString analysis was done with FACS-isolated Tregs from naive and nephritic FIR-tiger-*PD-L1*^−*/*−^ and FIR-tiger mice. Heatmap of immunological gene expression in Tregs is shown. (**C**) Relative gene expression levels of Tregs from naive and nephritic FIR-tiger-*PD-L1*^−*/*−^ and FIR-tiger mice are shown. Columns and bar graphs show mean values of three independent experiments. *p < 0.05; **p < 0.01; ***p < 0.001; ns, not significant; *Pdcd1*: PD-1; *Il2ra*: IL-2 receptor subunit alpha; *Entpd1*: CD39; *Gzmb*: granzyme B; *Csf1*: colony stimulating factor 1.

Having shown that PD-L1 deficiency did not result in impaired Treg differentiation or renal recruitment, we asked whether lack of PD-L1 instead compromises Treg function. To characterize more specifically the phenotype of Tregs in absence or presence of PD-L1 under homeostatic and inflammatory conditions, we FACS-sorted Tregs from naive and nephritic FIR-tiger-*PD-L1*^−*/*−^ and FIR-tiger mice and subjected these cells to a quantitative gene expression array profiling immunology-related genes (Fig. 5A). Interestingly, the gene expression pattern of Tregs from FIR-tiger-*PD-L1*^−*/*−^ mice was distinct from that of Tregs from FIR-tiger mice even in the steady state. They showed up-regulated expression of genes associated with cytokine/chemokine signaling, cell adhesion/migration and Treg function. To a lesser extent, we also found genes that were down-regulated in Tregs from FIR-tiger-*PD-L1*^−*/*−^ mice. Kidney inflammation altered gene expression of Tregs from nephritic FIR-tiger-*PD-L1*^−*/*−^ and FIR-tiger mice but again, there were strong differences in the gene expression profile of both Treg populations (Fig. 5B, Supplemental Fig. 6A,B). For instance, in contrast to Tregs from FIR-tiger mice, Tregs from FIR-tiger-*PD-L1*^−*/*−^ mice did not increase IL-10 expression during NTN. Interestingly, Tregs from naive and nephritic FIR-tiger-*PD-L1*^−*/*−^ mice showed up-regulated expression of IFNγ compared to Tregs from naive and nephritic FIR-tiger mice. We also found strongly elevated expression of PD-1 in the absence of PD-L1. Moreover, Tregs from nephritic FIR-tiger mice maintained high expression of the high-affinity IL-2Ra chain (*Il2ra*) during renal inflammation whereas Tregs from nephritic FIR-tiger-*PD-L1*^−*/*−^ mice showed decreased expression. We demonstrated increased expression of molecules associated with Treg function such as CD39 (*Entpd1*) and T-cell immunoreceptor with Ig and ITIM domains (*Tigit*) in the absence of PD-L1. In contrast, only Tregs from FIR-tiger mice showed enhanced expression of granzyme B (*GzmB*) during NTN. Lack of PD-L1 resulted in up-regulated expression of the chemokines CXCL10, CCL5, and XCL1, of the cytokine colony stimulating factor 1 (CSF1), and of the chemokine receptor CXCR3 (Fig. 5C). Thus, lack of PD-L1 profoundly altered the gene expression profile of Tregs in homeostasis and kidney inflammation.

### Impaired immunosuppressive capacity of Tregs in the absence of PD-L1

**Figure 6:**
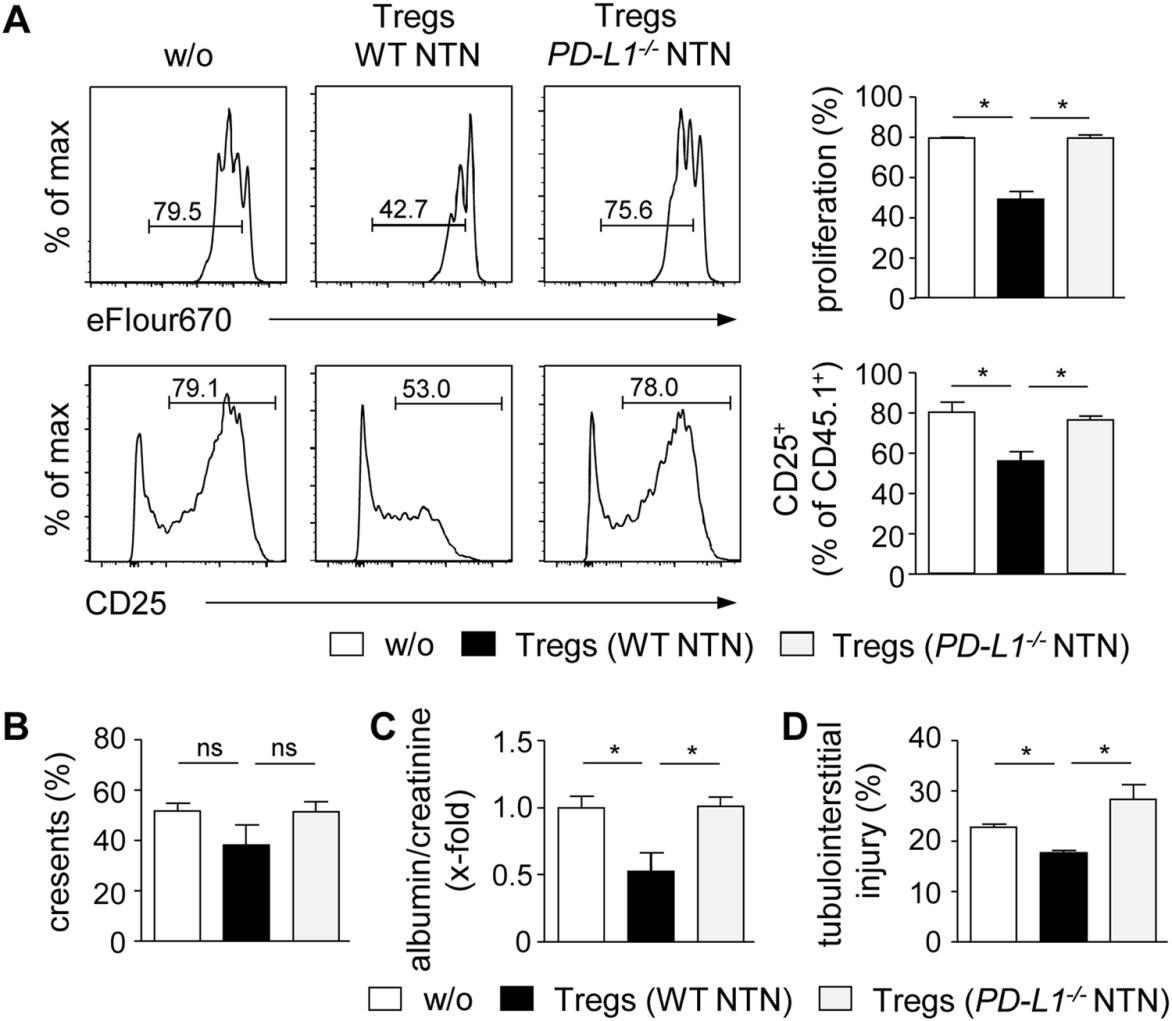
Impaired immunosuppressive function of Tregs from nephritic *PD-L1*^−*/*−^ mice. (**A**) Tregs from nephritic *PD-L1*^−*/*−^ and WT mice were co-cultured with CD4^+^ CD25^−^ T cells from CD45.1 mice in the presence of anti-CD3 antibody for three days. Responder CD4^+^ T-cell proliferation and activation were analyzed by flow cytometry. Representative dot plots are depicted. Mean ± SEM of one experiment out of three experiments are shown. (**B**) CD4^+^ CD25^−^ T cells were adoptively transferred into *Rag1*^−*/*−^ mice either alone or together with Tregs from nephritic *PD-L1*^−*/*−^ or WT mice. NTN was induced two days later and mice were analyzed 8 days after NTN induction. Crescent formation was quantified in PAS-stained kidney sections. (**C**) Albumin-creatinine-ratio was determined in urine by ELISA. (**D**) Tubulointerstitial injury was quantified in PAS-stained kidney sections. Mean ± SEM of two experiments with 5 mice per group are shown. *p < 0.05; ns, not significant; w/o: without Tregs.

To analyze Treg function *in vitro*, we performed suppression assays. Tregs were isolated from nephritic *PD-L1*^−*/*−^ and WT mice and co-cultured with eFlour670-labeld CD4^+^ CD25^−^ responder T cells from CD45.1 mice in the presence of anti-CD3 antibody for 3 days. Tregs from nephritic WT mice strongly reduced proliferation and activation of responder CD4^+^ T cells. In contrast, Tregs from nephritic *PD-L1*^−*/*−^ mice failed to suppress responder CD4^+^ T-cell proliferation and activation (Fig. 6A). By performing adoptive cell transfer experiments, we analyzed the suppressive capacity of Tregs *in vivo*. FACS-sorted CD4^+^ CD25^−^ T cells were i.v. injected either alone or together with Tregs from nephritic *PD-L1*^−*/*−^ or WT mice into *Rag1*^−*/*−^ mice. Two days later, NTN was induced. Tregs from nephritic WT mice did not significantly alter crescent formation (Fig. 6B) but improved kidney function demonstrated by reduced proteinuria (Fig. 6C). Beside glomerular damage, tubulointerstitial damage also contributes to impairment of renal function in kidney disease and we showed that Tregs from nephritic WT mice ameliorated tubulointerstitial injury during NTN (Fig. 6D). In contrast, Tregs from nephritic *PD-L1*^−*/*−^ mice had no suppressive effect on disease pathogenesis of NTN (Fig. 6B–D) indicating impaired immunosuppressive function of Tregs in the absence of PD-L1.

## Discussion

Crescentic glomerulonephritis is a life-threatening disease triggered by so far poorly defined mechanisms. Thus, identifying molecular and cellular pathways involved in the pathogenesis of cGN is of high clinical relevance. In this study, we showed that the co-inhibitory PD-1/PD-L1 pathway contributes to immune regulation in cGN by promoting Treg-mediated suppression of the inflammatory Th1 immune response.

During inflammation, lymphocyte activation has to be regulated in order to dampen ongoing immune responses and to prevent autoimmunity. The crucial immunoregulatory function of Tregs in kidney disease was demonstrated in various studies and are reviewed elsewhere^24^. We found that the frequency of Tregs expressing PD-L1 was increased during NTN. There is some evidence in the literature that Tregs mediate immunosuppression by PD-L1-driven lymphocyte inhibition. Blockage of the PD-1/PD-L1 pathway by using an anti-PD-L1 antibody was shown to inhibit Treg-mediated suppression of T-cell activation *in vitro*^25,26^. Moreover, antigen-specific suppression of autoreactive B cells by PD-L1-expressing Tregs was demonstrated in a mouse model with glomerular model antigen expression^27^. In our study, we showed an elevated renal Th1 immune response as well as IgG antibody response in nephritic *PD-L1*^−*/*−^ mice possibly due to lack of PD-L1 expression by Tregs.

In contrast to a previous study, which linked PD-1/PD-L1 signaling to Treg induction^3^, we found that lack of PD-L1 did not affect development of Foxp3^+^ Tregs. The number of Tregs was even increased in the inflamed kidneys of FIR-tiger-*PD-L1*^−*/*−^ mice. The ability of Tregs to access inflammatory tissue is regulated by expression of receptors for chemokines produced in the inflamed organ^28^. We showed elevated gene expression of the chemokine receptor CXCR3 by Tregs from nephritic FIR-tiger-*PD-L1*^−*/*−^ mice and together with enhanced renal expression of the corresponding CXCR3 ligand CXCL10, this might account for increased recruitment of Tregs in the absence of PD-L1. This assumption is supported by a study showing that Treg-specific deletion of CXCR3 resulted in reduced renal infiltration of Tregs during NTN and an exaggerated Th1 immune response^29^. However, despite elevated Treg numbers in the inflamed kidney, lack of PD-L1 aggravated disease pathogenesis of NTN and was associated with an amplified renal Th1 immune response. Since we have shown previously that Tregs control the Th1 immune response during NTN^20^, these findings suggest an impaired immunosuppressive function of Tregs in the absence of PD-L1. Indeed, we showed that Tregs from nephritic *PD-L1*^−*/*−^ mice failed to inhibit CD4^+^ T-cell activation or to suppress disease pathology of NTN. One mechanism by which Tregs mediate immunosuppression during NTN is by production of the anti-inflammatory cytokine IL-10^21^. Gene expression analysis revealed that in the absence of PD-L1, Tregs did not up-regulate expression of IL-10 during kidney inflammation. Moreover, they down-regulated gene expression of the high affinity IL-2Ra chain. Since IL-2/IL-2R signaling has been shown to be essential for Treg function and fitness^30^, this might affect immunosuppressive capacity of Tregs during NTN. It has been further shown that PD-1-deficient Tregs exhibit a more suppressive phenotype than PD-1^+^ Tregs^31^. Moreover, PD-1 expression by Tregs of viral hepatitis patients inhibited their immunosuppressive function^32^. Since Tregs from nephritic *PD-L1*^−*/*−^ mice up-regulated expression of PD-1, this might be another reason for their impaired immunosuppressive capacity. Tregs also promote immunosuppression by inducing PD-L1 expression on DCs, which in turn suppress T-cell activation as demonstrated in graft-versus-host disease^33^. In the absence of PD-L1, DCs do not acquire the ability to promote tolerance by this mechanism. We even found that DCs from nephritic *PD-L1*^−*/*−^ mice exhibit a more activated phenotype with elevated MHCII expression that could drive T-cell activation and polarization during NTN.

Increased frequencies of Th1 cells in the kidneys of nephritic *PD-L1*^−*/*−^ mice might also be caused by elevated renal expression of the Th1 cell-attracting chemokine CXCL10 whose expression is further triggered by IFNγ. This assumption was supported by the finding that inhibition of IFNγ in nephritic *PD-L1*^−*/*−^ mice reduced CXCL10 expression and renal recruitment of Th1 cells. Since blockage of IFNγ also ameliorated NTN, we conclude that elevated IFNγ levels in the absence of PD-L1 drive disease pathogenesis. Enhanced IFNγ-mediated immune responses were shown in other diseases caused by a nonfunctional PD-1/PD-L1 pathway. For example, increased disease severity of EAE in *PD-1*^−*/*−^ mice was linked to a higher frequency of IFNγ-expressing T cells^34^. In graft-versus-host disease, rapid mortality of *PD-L1*^−*/*−^ mice was associated with elevated Th1 cytokine production^35^, and in heart transplantation, absence of PD-L1 promoted Th1-cell polarization and allograft rejection^36^ strongly indicating the essential role of the PD-1/PD-L1 pathway in controlling Th1 immune responses in autoimmune diseases.

The immunosuppressive function of the PD-1/PD-L1 pathway was demonstrated in several autoimmune diseases. *PD-1*^−*/*−^ mice developed lupus-like glomerulonephritis, destructive arthritis, and dilated cardiomyopathy^9,37^. Blockage of PD-1/PD-L1 interaction aggravated diabetes in nonobese diabetic mice^7,38^, accelerated experimental autoimmune encephalomyelitis (EAE)^34^, and worsened disease pathogenesis of accelerated NTN^11^. We confirmed the pathological findings of the study by Menke *et al*. by using FIR-tiger-*PD-L1*^−*/*−^ and *PD-L1*^−*/*−^ mice and by blockage of PD-L1 in WT mice. The authors also showed that PD-L2^−*/*−^ mice developed more severe accelerated NTN indicating an immunoregulatory function for the second PD-1 ligand in kidney inflammation^11^. However, renal expression of PD-L2 was not altered in nephritic FIR-tiger-*PD-L1*^−*/*−^ and *PD-L1*^−*/*−^ mice and despite up-regulated expression of PD-1, PD-L2 did not compensate the absence of PD-L1.

Several therapeutic strategies activating the PD-1/PD-L1 pathway in autoimmune disease have been tested in mice. One study reported that overexpression of PD-L1 on DCs reduced severity of EAE^39^. Systemic activation of the PD-1/PD-L1 pathway by injection of a PD-L1/Fc fusion protein ameliorated progression of intracerebral hemorrhage^40^, colitis^41^, and collagen-induced arthritis^42^. In kidney disease, administration of a recombinant adenovirus encoding PD-L1 attenuated lupus-like nephritis^43^, and treatment with a PD-L1/Fc fusion protein ameliorated experimental autoimmune glomerulonephritis in rats^44^. In contrast, inhibition of the PD-1/PD-L1 pathway by administration of the checkpoint inhibitors pembrolizumab or nivolumab during cancer immunotherapy caused a higher incidence of acute interstitial nephritis in patients with melanoma or lung cancer^45^. The results of our study illustrate the important immunoregulatory function of the PD-1/PD-L1 pathway in NTN by ensuring proper Treg function in renal inflammation. Therefore, stimulating this pathway may provide a therapeutic option in cGN.

## Methods

### Animals

C57BL/6, *PD-L1*^−*/*−^, *Rag1*^−*/*−^, CD45.1, FIR-tiger (Foxp3-IRES-mRFP [FIR] x IL-10-IRES-GFP [tiger])^22^, and FIR-tiger-*PD-L1*^−*/*−^ mice were bred in the animal facility of the University Medical Center Hamburg-Eppendorf (Hamburg, Germany). A detailed genotyping protocol of FIR-tiger-*PD-L1*^−*/*−^ mice and analysis of PD-L1 expression in these mice is shown in Supplemental Figs 2 and 3. FIR-tiger mice and *PD-L1*^−*/*−^ mice were provided by Richard Flavell (Yale University, New Haven, CT) and Lieping Chen (Yale University, New Haven, CT), respectively. Mouse experiments were conducted according to the German animal protection law and approved by the institutional review board (Behörde für Gesundheit und Verbraucherschutz, Hamburg, Germany; G20/13). Mice received humane care according to the national guidelines of the National Institutes of Health in Germany.

### Animal treatment and functional studies

NTN was induced in male mice (8–12 weeks old) by intraperitoneal (i.p.) injection of 2.5 mg nephrotoxic sheep serum per gram of mouse body weight as described previously^46^. Mice were sacrificed 8 days after NTN induction. To assess systemic antibody response, heart blood was drawn from individual mice. One day before killing, mice were housed in metabolic cages for urine collection. Albuminuria was determined by ELISA (Mice-Albumin Kit; Bethyl, Montgomery, TX). C57BL/6 mice were treated i.p. with an anti-PD-L1 antibody (250 µg/mouse; 10 F.9G2; BioXCell, West Lebanon, NH) daily starting one day after NTN induction. As isotype control, mice received rat IgG2b (250 µg/mouse; LTF-2; BioXCell). *PD-L1*^−*/*−^ mice received i.p. an anti-IFNγ antibody (250 µg/mouse; XMG1.2; BioXCell) one day before and 4 days after NTN induction. As isotype control, rat IgG1 (1 mg/mouse; HRPN; BioXCell) was injected.

### Histological analysis

Immunohistochemistry was performed by routine procedures as described previously^47^. Crescent formation was assessed in 30 glomeruli/mouse in a blinded fashion in PAS-stained, paraffin-embedded kidney sections.

### Antigen-specific humoral immune response

Mouse anti-sheep IgG antibody titers were measured by ELISA using sera collected 8 days after NTN induction. In brief, ELISA microtiter plates were coated with 100 µl sheep IgG (100 µg/ml; Sigma-Aldrich, St. Louis, MO) in carbonate-bicarbonate buffer overnight at 4 °C. After blocking with 1% BSA in TBS, the plates were incubated with serial dilutions of mouse serum for one hour at room temperature. Bound mouse IgG was detected using peroxidase-conjugated goat anti-mouse IgG (Biozol, Eching, Germany), TMB peroxidase substrate (Thermo Fisher Scientific, Waltham, MA), and absorbance readings (at 450 nm). Lack of cross-reactivity of the secondary antibody with sheep IgG was demonstrated by omitting the primary antibody. Bound mouse immunoglobulin isotypes were detected using peroxidase-conjugated rabbit anti-mouse IgG (total), IgG1, IgG2a/c, and IgG2b antibodies (Zymed-Invitrogen, Karlsruhe, Germany).

### Isolation of renal cells and splenocytes

Kidneys and spleens were harvested from nephritic mice 8 days after NTN induction. Splenic tissue was passed through 70 µm nylon meshes prior to erythrocyte lysis using NH_4_Cl. Kidneys were finely minced and digested for 40 min at 37 °C with 0.4 mg/ml collagenase D (Roche, Mannheim, Germany) and 0.01 mg/ml DNase I (Roche) in RPMI 1640 supplemented with 10% heat-inactivated fetal calf serum (Thermo Fisher Scientific). Erythrocytes were lysed with NH_4_Cl and cell suspensions were sequentially filtered through 70 µm and 40 µm nylon meshes and washed with Ca^2+^- and Mg^2+^-free HBSS (Thermo Fisher Scientific).

### Flow cytometry

Renal cells and splenocytes were incubated with anti-CD16/32 antibody (93; BioLegend, San Diego, CA) prior to antibody staining to prevent unspecific binding. LIVE/DEAD Fixable Red Dead Cell Stain Kit (Thermo Fisher Scientific) was used to exclude dead cells. Cells were surfaced-stained with fluorochrome-labeled antibodies specific to CD3 (AF700; 17A2), PD-1 (FITC; J43), PD-L1 (PE; MIH5; all eBioscience, San Diego, CA), CD4 (BV711; 13B8.2), CD45.1 (A20; all BD Biosciences, San Jose, CA), CD45 (PerCP, BV570; 30-F11), CD11b (AF700; M1/70), CD11c (BV605; N418), Ly-6C (PerCP; HK1.4), MHCII (FITC; M5/114.15.2), TCRβ (PE-Cy7; H57-597), CD25 (PE; PC-61), Nrp-1 (PE; 3E12), Helios (FITC; 22F6), CD44 (BV421; IM7), CD62L (PerCP-Cy5.5; MEL-14; all BioLegend). For intracellular cytokine staining, cells were stimulated with phorbol myristate acetate (10 ng/ml) and ionomycin (250 ng/ml) for 5 h with the addition of brefeldin A (1 µg/ml; all Sigma Aldrich) after 30 min. After surface and Live/Dead staining, cells were fixed in Fix/Perm solution before incubation in Perm/Wash buffer (both BD Biosciences) with antibodies specific to IFNγ (APC; XMG1.2; eBioscience) and IL-17A (V450; TC11-18H10; BD Pharmingen). For intranuclear staining, cells were fixed using the Transcription Factor Staining Buffer Set (eBioscience) and incubated in Permeabilization buffer with an antibody specific to Foxp3 (AF647; MF-14; BioLegend). Flow cytometry data were analyzed by using fluorescence minus one controls.

### Antigen-specific cellular immune response

Splenocytes were cultured at a density of 4 × 10^6^ cell/ml in RPMI 1640 medium supplemented with 10% fetal bovine serum, 1% penicillin/streptomycin, 1% sodium pyruvate, and 0.1% β-mercaptoethanol (all Gibco/ThermoFisher Scientific) in the presence of 20 µg/ml sheep IgG for three days. Cytokine levels were determined in culture supernatants using 96-well high-binding flat-bottom microtiter plates (Greiner Bio-One, Frickenhausen, Germany) and Duo Set ELISA for IFNγ (BioLegend, San Diego, CA) according to the manufacturer’s protocol.

### Suppression assay

Tregs were isolated from *PD-L1*^−*/*−^ and WT mice 8 days after induction of NTN. Therefore, CD4^+^ T cells were enriched using the CD4^+^ T Cell Isolation Kit (Miltenyi Biotec, Bergisch Gladbach, Germany). Subsequently, CD4^+^ CD25^hi^ Tregs were sorted by FACS (BD FACSAriaTM III sorter, BD Biosciences). CD4^+^ CD25^−^ responder T cells from CD45.1 mice were isolated by FACS and labeled with Cell Proliferation Dye eFlour670. Then, 1 × 10^5^ responder T cells were co-cultured with 1 × 10^5^ Tregs in the presence of an anti-CD3 antibody (145-2C11; BioLegend) for 3 days.

### Adoptive transfer experiments

CD4^+^ CD25^hi^ Tregs were sorted by FACS from nephritic *PD-L1*^−*/*−^ and WT mice. 1 × 10^6^ FACS-sorted CD4^+^ CD25^−^ T cells from CD45.1 mice were adoptively transferred into *Rag1*^−*/*−^ mice either alone or in combination with 5 × 10^5^ Tregs from nephritic *PD-L1*^−*/*−^ or WT mice. After 2 days, NTN was induced and mice were analyzed 8 days later.

### Quantitative real-time RT- PCR analysis

Total RNA was isolated from shock-frozen kidney tissue using the NucleoSpin RNA Kit (Machery-Nagel, Duren, Germany) according to the manufacturer’s instruction. RNA was transcribed into cDNA using the Verso cDNA Synthesis Kit (Life Technologies, Carlsbad, CA) on a MyCycler thermal cycler (BioRad, München, Germany). Quantitative RT-PCR was performed using the ABsolute qPCR SYBR Green Mix (Thermo Scientific). The relative mRNA levels were calculated using the ∆∆CT method after normalization to the reference genes β-actin or 18 S. Quantification was shown in x-fold changes to the corresponding control cDNA. Primers were obtained from Metabion (Martinsried, Germany). Sequences of the primers are listed in Supplemental Table 1.

### Treg gene expression analysis

Foxp3^+^ Tregs were isolated from spleen and lymph nodes of naive and nephritic FIR-tiger and FIR-tiger-*PD-L1*^−*/*−^ mice. Therefore, CD4^+^ T cells were enriched from single cell suspensions by using the CD4^+^ T Cell Isolation Kit (Miltenyi Biotec). Thereafter, CD4^+^ Foxp3^+^ (mRFP^+^) Tregs were purely isolated by FACS. RNA of 1–2 × 10^6^ Tregs was isolated by the RNeasy Mini Kit (Quiagen) and digested with RNase-free DNase I (Quiagen) according to the manufacturer’s instructions. Gene expression analysis was performed using the NanoString assay with the nCounter Immunology Panel profiling 561 immunology-related genes **(**NanoString Technologies, Hamburg, Germany) according to the manufacturer’s instructions. The results were analyzed with the nSolver Analysis Software 2.5 (NanoString Technologies). Data were calculated in x-fold changes compared to the corresponding control mRNA and displayed as heat map using the web server Heatmapper^48^. Clustering was done using the average linkage and the Pearson distance.

### Statistical analyses

Data were analyzed using the GraphPad Prism software (GraphPad software, San Diego, CA). Statistical comparison was carried out using the Mann-Whitney U test or the one-way ANOVA with post analysis by Tukey-Kramer test. Data were expressed as means ± SEM. A p value of less than 0.05 was considered statistically significant with the following ranges *p < 0.05, **p < 0.01, and ***p < 0.001.

## Supplementary information

Dataset 1

## Data Availability

The datasets generated during and/or analyzed during the current study are available from the corresponding author on reasonable request.
