# Supplementary material for: The co-inhibitory molecule PD-L1 contributes to regulatory T cell-mediated protection in murine crescentic glomerulonephritis
Source: Sci Rep. 2019 Feb 14;9:2038. doi: 10.1038/s41598-018-38432-3 (PMC6375967; doi:10.1038/s41598-018-38432-3)
Supplement: Supplementary file 1 — Dataset 1 [file 41598_2018_38432_MOESM1_ESM.pdf]

The co-inhibitory molecule PD-L1 contributes to regulatory T cell-mediated protection in murine crescentic glomerulonephritis

Katrin Neumann, Annett Ostmann, Philippe Christophe Breda, Aaron Ochel, Frank Tacke, Hans-Joachim Paust, Ulf Panzer, Gisa Tiegs

Table 1. Sequences of the primer used for analysis of mRNA expression.

**Target**	**Forward primer**<br>**Reverse primer**	**Amplicon length**	**Annealing temperature**
β-actin	5´-TGGAATCCTGTGGCATCCATGAAA-3´<br>3´-TAAAACGCAGCTCAGTAACAGTCCG-5´	348 bp	56°C
IFNγ	5´-GAACGCTACACACTGCATC-3´<br>3´-GAGCTCATTGAATGCTTGG-5´	390 bp	56°C
IL-12p40	5´-AGGTCACACTGGACCAAAGG-3´<br>3´-TGGTTTGATGATGTCCCTGA-5´	173 bp	60°C
T-bet	5´-ACCTGGACCCAACTGTCAAC-3´<br>3´-AACTGTGTTCCCGAGGTGTC-5´	158 bp	60°C
CXCL10	5´-GCCGTCATTTTCTGCCTCAT-3´<br>3´-TGCAGCGGACCGTCCTT-5´	76 bp	60°C
IL-17A	5´-TCCAGAAGGCCCTCAGACTA-3´<br>3´-ACACCCACCAGCATCTTCTC-5´	248 bp	60°C
IL-6	5´-GATGGATGCTACCAAACTGGA-3´<br>3´-GGAAATTGGGGTAGGAAGGA-5´	222 bp	60°C
RORγt	5´-GAGCCAAGTTCTCAGTCATGAG-3´<br>3´-GGCCAAACTTGACAGCATCT-5´	265 bp	62°C
Foxp3	5´-GGCCCTTCTCCAGGACAGA-3´<br>3´-GCTGATCATGGCTGGGTTGT-5´	112 bp	57°C
PD-L1	5´-CTGCCAAAGGACCAGCTTTT-3´<br>3´-GGCTGGATCCACGGAAATTC-5´	180 bp	59°C
PD-L2	5´-TGGGGAGATCTGGGGAAGTA-3´<br>3´-TTTTGCCAGGACACTTCTGC-5´	190 bp	59°C
PD-1	5´-GGAGCAGAGCTCGTGGTAAC-3´<br>3´-GCTCCTCCTTCAGAGTGTCG-5´	229 bp	63°C

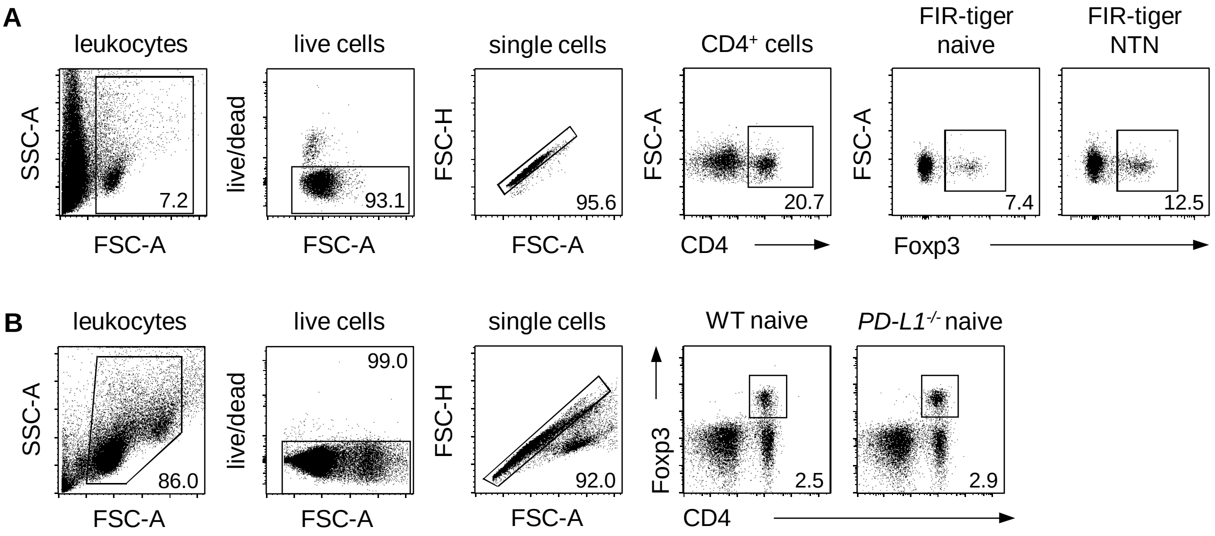

Supplemental Fig. 1. Gating strategies used in flow cytometry analysis to detect Foxp3$^+$ Tregs. (A) NTN was induced in FIR-tiger mice by i.p. injection of the nephritogenic NTN serum and mice were analyzed 8 days later. Renal cells of naive and nephritic FIR-tiger mice were stained with a live/death dye and an anti-CD4 antibody. Subsequently, living single CD4$^+$ cells were analyzed for expression of Foxp3 (mRFP). (B) Splenic cells of naive *PD-L1*$^{-/-}$ and WT mice were stained with a live/death dye and antibodies directed against CD4 and Foxp3. The frequency of living single CD4$^+$ Foxp3$^+$ Tregs was determined. Representative dot plots of one experiment out of two experiments with 4 mice per group are shown.

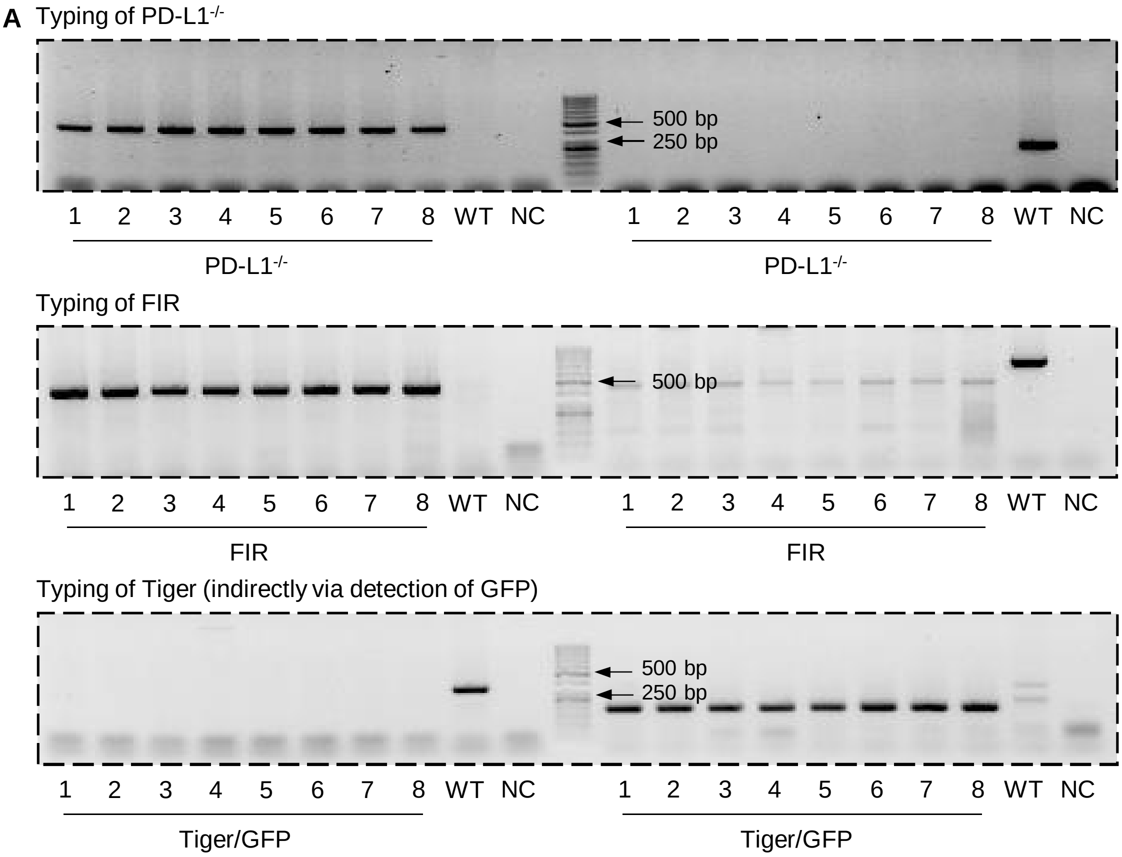

B

Gene	Sequence	PRC product
PD-L1	P1: AGAACGGGAGCTGGACCTGCTTGCGTTAG<br>P2: ATTGACTTTCAGCGTGATTCGCTTGTAG<br>P3: TTCTATCGCCTTCTTGACGAGTTCTTCTG	Knockout: 450 bp (P2+P3)<br>WT: 250 bp (P1+P2)
FIR	Fir1: CAAAACCAAGAAAAGGTGGGC<br>Fir2: GGAATGCTCGTCAAGAAGACAGG<br>Fir3: CATCTTGGAGAGTCGGTGTG	Transgen: 470 bp (Fir1+Fir2)<br>WT: 692 bp (Fir1+Fir3)
Tiger/ GFP	WT1: GTGTGTATTGAGTCTGCTGGAC<br>WT2: GTGTGGCCAGCCTTAGAATAG<br>WT3: GGTTGCCTTGACCATCGATG<br>GFP1: AAGTCGTGCTGCTTCATGTG<br>GFP2: ACGTAAACGGCCACAAGTTC	GFP: 200 bp (GFP1+GFP2)<br>WT: 350 bp (WT1-3)

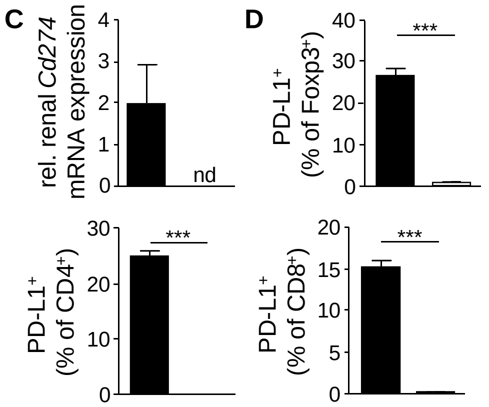

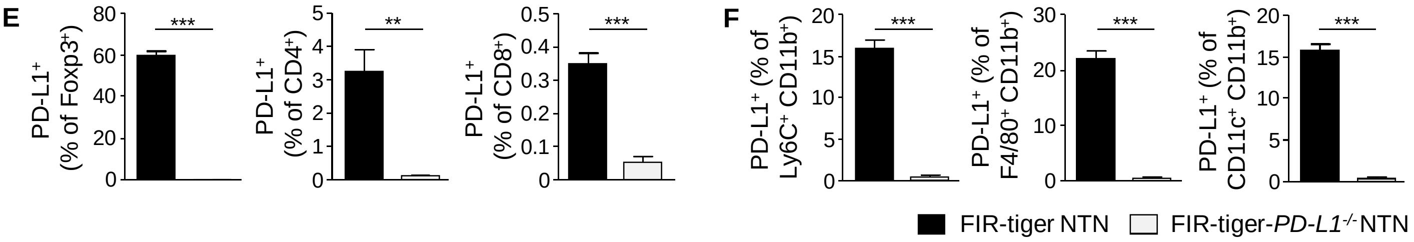

Supplemental Fig. 2. Genotyping of Fir-tiger-*PD-L1*$^{-/-}$ mice and expression of PD-L1 by these mice. (A) Eight FIR-tiger-*PD-L1*$^{-/-}$ mice and one WT mice were used for genotyping. Dividing lines mark cropped areas of the gels. Original gels of the typing PCRs are shown in Supplemental Fig. 3. (B) Primer sequences and PCR product size of each analyzed gene are shown. (C) Renal mRNA expression of nephritic FIR-tiger and FIR-tiger-*PD-L1*$^{-/-}$ mice was analyzed by quantitative real-time RT-PCR and normalized to the reference gene β-actin. (D) Frequencies of renal and (E) splenic PD-L1-expressing T-cell populations as well as (F) APC populations were analyzed by flow cytometry. FIR: Foxp3-IRES-mRFP, Tiger: IL-10-IRES-GFP, WT: wildtype, NC: negative control, GFP: green fluorescent protein, bp: base pairs, nd: not detectable

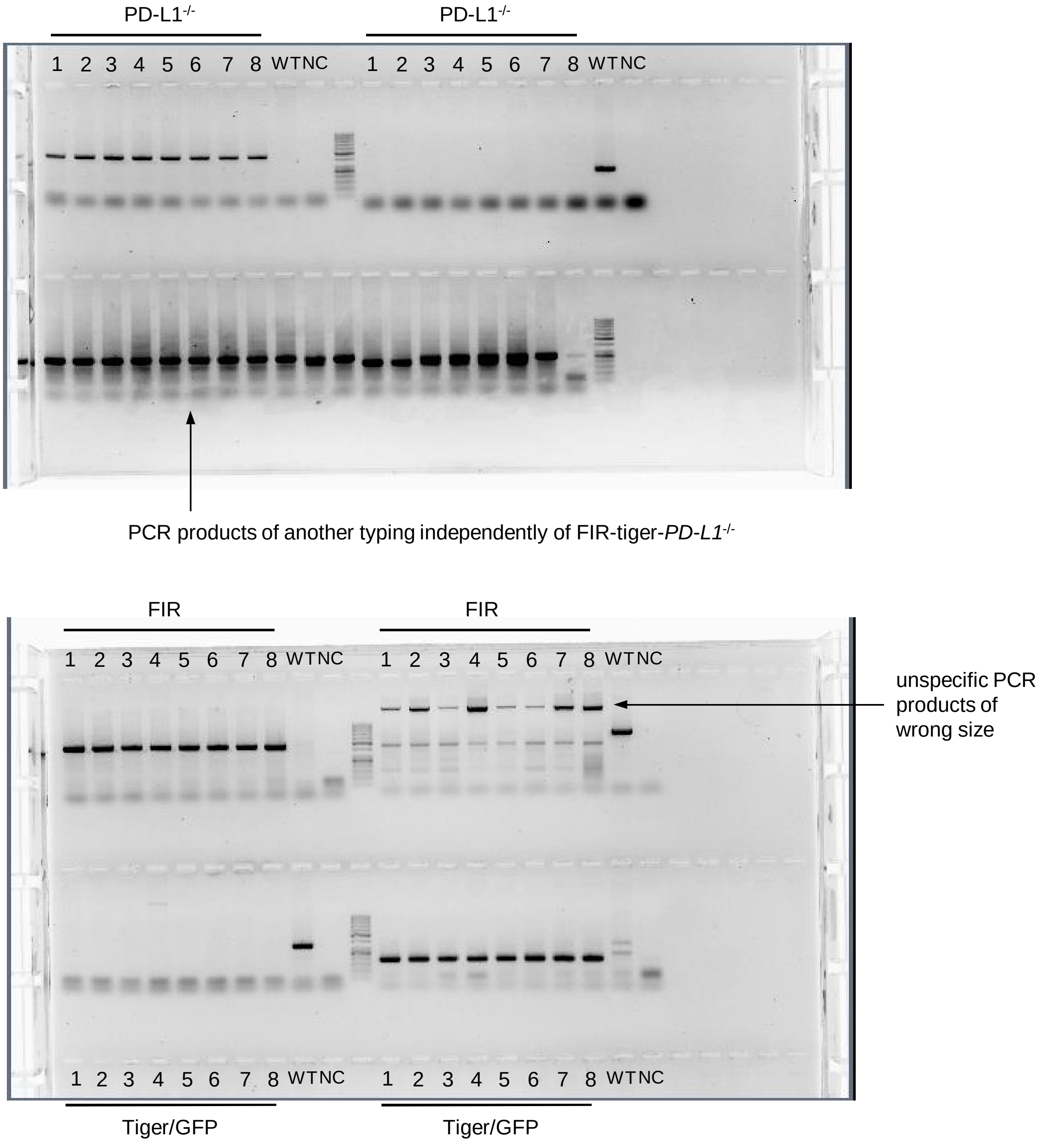

Supplemental Fig. 3. Genotyping of FIR-tiger-*PD-L1*$^{-/-}$ mice. Eight FIR-tiger-*PD-L1*$^{-/-}$ mice and one WT mice were used for genotyping. Original gels are shown.

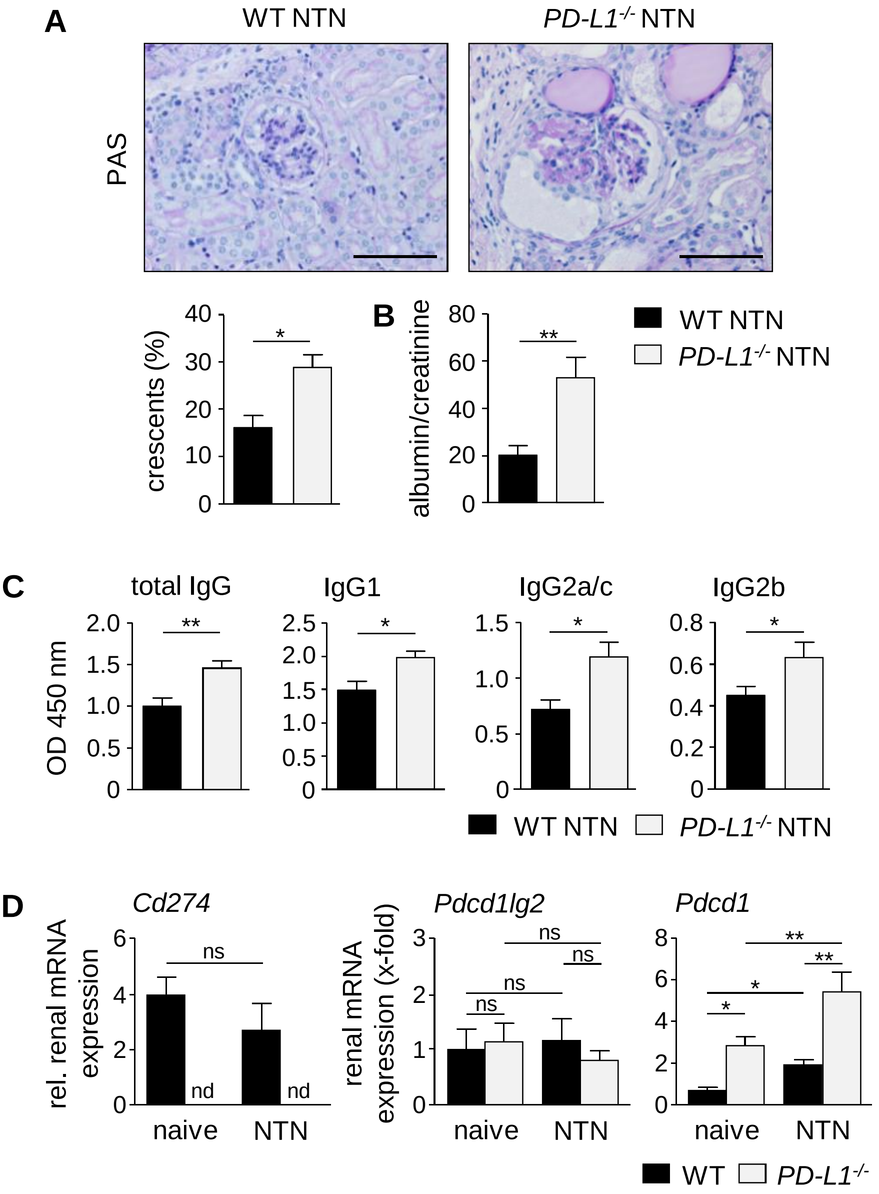

Supplemental Fig. 4. Exacerbated NTN in nephritic *PD-L1$^{-/-}$* mice. NTN was induced in *PD-L1$^{-/-}$* and WT mice by i.p. injection of the nephritogenic NTN serum and mice were analyzed 8 days later. (A) Glomerular crescent formation was quantified in PAS-stained kidney sections of nephritic *PD-L1$^{-/-}$* and WT mice. (B) Renal dysfunction was assessed by determination of the albumin-creatinine-ratio in urine by ELISA. (C) Levels of mouse anti-sheep total IgG and isotypes of IgG1, IgG2a/c, and IgG2b were determined in serum of nephritic *PD-L1$^{-/-}$* and WT mice by ELISA. (D) Renal mRNA expression of naive and nephritic *PD-L1$^{-/-}$* and WT mice was analyzed by quantitative real-time RT-PCR and normalized to mRNA expression of naive WT mice. Representative photomicrographs were shown. Scale bar represents 100 µm. Mean SEM of one experiment out of 3 experiments with 5-6 mice per group are shown. *p< 0.05; **p< 0.01; ns, not significant; nd, not detectable; *Cd274*: PD-L1; *Pdcd1lg2*: PD-L2; *Pdcd1*: PD-1

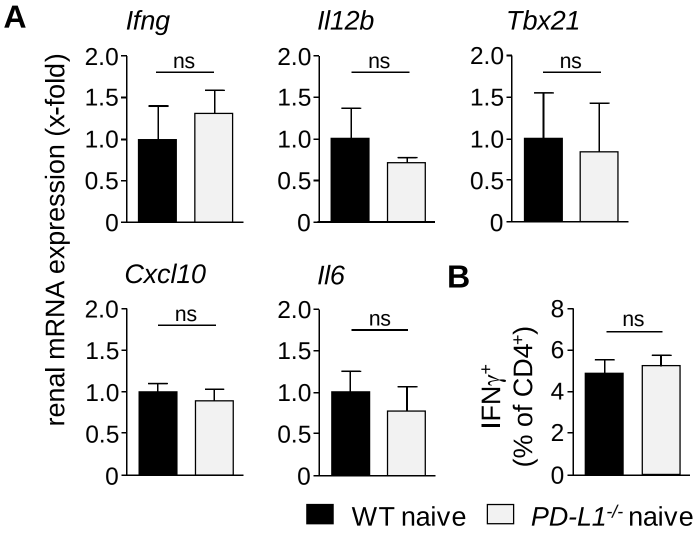

Supplemental Fig. 5. Phenotype analysis of naive *PD-L1*$^{-/-}$ and WT mice. (A) Renal mRNA expression of naive *PD-L1*$^{-/-}$ mice was analyzed by quantitative real-time RT-PCR and normalized to mRNA expression of naive WT mice. (B) Frequency of renal IFNγ^{+} CD4^{+} T cells was determined in naive *PD-L1*$^{-/-}$ and WT mice by flow cytometry. Mean ± SEM of 6 mice per group are shown. ns, not significant; *Il12b*: IL-12 subunit p40; *Tbx21*: T-bet; *Rorc*: RORγt

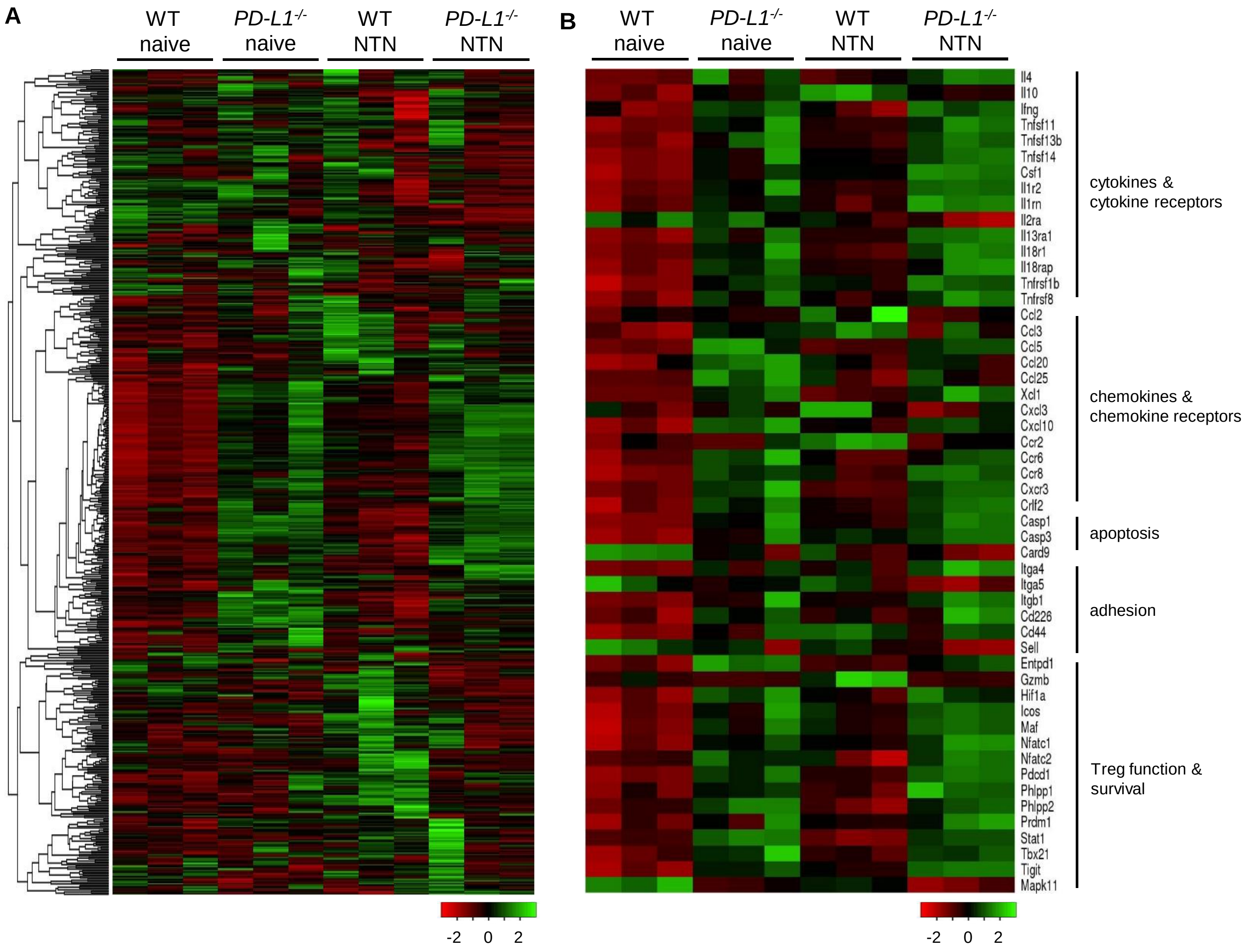

Supplemental Fig. 6. Gen expression profile of Tregs in absence and presence of PD-L1. Foxp3$^+$ Tregs were isolated by FACS from naive and nephritic FIR-tiger (WT) mice and FIR-tiger-*PD-L1*$^{-/-}$ (*PD-L1*$^{-/-}$) mice. NanoString analysis was performed with the nCounter Immunology Panel profiling 561 immunology-related genes. (A) Heatmap of immune-gene expression in Tregs from *PD-L1*$^{-/-}$ and WT mice is shown. (B) Heatmap of genes differentially expressed in Tregs from *PD-L1*$^{-/-}$ and WT mice is depicted. Heatmaps show data of three independent experiments.
